# Investigation of Fire Effects on Reinforced Concrete Members via Finite Element Analysis

**DOI:** 10.1021/acsomega.2c03414

**Published:** 2022-07-20

**Authors:** Betül Aliş, Casim Yazici, Fatih Mehmet Özkal

**Affiliations:** † M.Sc., Department of Civil Engineering, Atatürk University, 25240 Erzurum, Turkey; ‡ Ph.D. Candidate, Department of Construction, Ağrı İbrahim Çeçen University, 04400 Ağrı, Turkey; § Ph.D., Associate Prof., Department of Civil Engineering, Atatürk University, 25240 Erzurum, Turkey

## Abstract

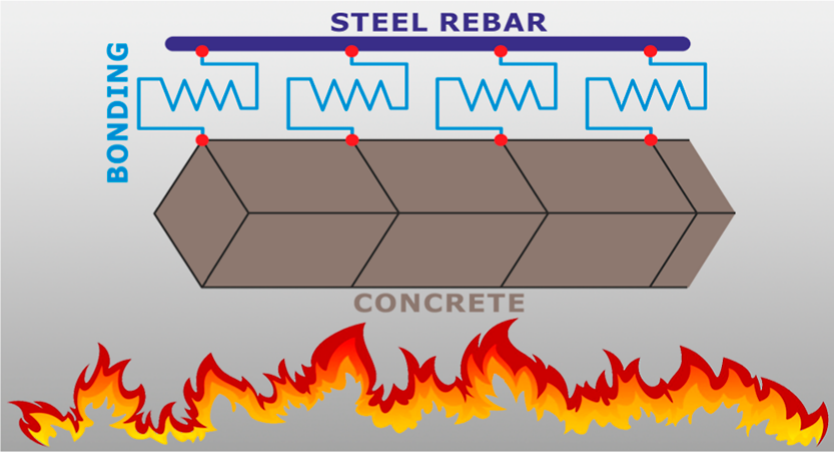

Structural deterioration during fire leads to significant economic losses, severe injuries, and deaths. Research to accurately estimate the impact of fire on structural security and performance, and to identify ways to reduce it, has been increasing recently with capital investments in the building and infrastructure sectors. This research aims to establish a reliable algorithm for simulating the behavior of reinforced concrete (RC) beams under thermal and structural loads. The proposed algorithm is based on the combination of thermal and structural analyses using the sequential link technique. These analyses use material characteristics such as conductivity, specific heat, stress–strain relationship, and thermal expansion to capture thermal and structural responses during the heating phases according to Eurocode 1 and Eurocode 2 using the finite element method. Beam models in the study, which have been exposed to the ISO-834 fire curve, were designed to exhibit flexural failure. Nonlinear numerical analysis results have mostly coincided with the previous studies regarding the residual load-bearing capacity. Depending on the outcomes of the previous experimental studies, an RC member’s structural strength increases when the internal temperature is between 150 and 250 °C and degradation starts after 300 °C. This outcome has been supported by the previous numerical and experimental studies, propounding the accuracy of preferred modeling and analysis approaches. As the essential distinctness of the research, the effects of elevated temperatures on the bonding behavior between concrete and rebar were considered for numerical analyses.

## 1 Introduction

Structural deterioration is seen during many fire events every year. It is known that such events lead to severe injuries, deaths, and significant economic loss. Among the factors affecting the amount of damage and losses caused by fires, type of structures and fire resistance and durability of the materials are essential. Concrete has the advantage of being resistant to fire, as it is a non-flammable material with low thermal conductivity.^1^ When the outer surface of a concrete element is exposed to high temperatures, the inner part of the concrete maintains its load-carrying capacity, and its low thermal conductivity protects the steel reinforcements from high temperatures. However, concrete resistance to fire should be considered at the design stages, as its strength deteriorates when exposed to high temperatures.^2^ Although concrete and steel are non-flammable materials, it has been recorded that concrete has a loss of strength at high temperatures.^3−6^

Laboratory experiments aiming to evaluate fire resistance are expensive, time-consuming, and there are limitations to test different parameters. Using numerical models to evaluate the structural fire resistance of reinforced concrete (RC) members is an alternative to the experiments. Numerical models enable researchers to take various parameters into account accurately and cost-effectively. This study presents a three-dimensional (3D) finite element (FE) model accurately estimating the thermal and mechanical behavior of reinforced concrete beams exposed to fire. This finite element analysis approach highly considers modeling the bonding behavior between reinforcement bars and concrete, a feature rarely considered in previous numerical studies. It showed that the inclusion of rebar-concrete bonding (adherence) yields more accurate results regarding the behavior of RC members exposed to fire.

This study is based on establishing a modeling approach to provide ideas and information about the residual strength of RC members, which are intended to be re-used after a fire. Numerical outcomes presented in the research demonstrate that finite element analysis of RC members can be used directly in performance-based fire safety design and parametric studies aiming to develop simple design rules.

It has been intended to achieve a reliable algorithm to simulate reinforced concrete beams under both mechanical and fire loads in this study. The proposed approach was modeled by ANSYS software for 3D analysis of RC members under high temperatures, and their thermal and structural analyses were performed. Most numerical analysis software such as ANSYS uses the sequential combination technique or the direct link technique to interconnect two types of analyses. The sequential combination technique is based on the concept of running each type of analysis separately and applying the results of the first analysis as initial conditions for the second one. On the other hand, the direct connection technique has been used as a single combined analysis for thermal and structural analyses with the activated degrees of freedom at each node.

## 2 Review of the Previous Research

Ellingwood and Lin^7^ stated that the temperature in steel rebars is the most critical factor affecting the strength of beams, while examining the bending effect of RC beams in case of being exposed to ASTM E119 standard fire. In their studies on RC members, Kumar and Kumar^8^ and Chen et al.^9^ showed that the increased load-carrying capacity decreases due to the increased exposure time to fire. Ahmad et al.^10^ and Sharma et al.^11^ found that the strength of concrete decreases with increasing temperature, and 600 °C is the critical temperature for serious deterioration in concrete.

Exposure of RC members to high temperatures causes significant losses on the mechanical and physical properties of concrete and steel reinforcements and also their bonding performance. Deterioration of bonding features in a fire can significantly affect the load capacity of concrete members. For this reason, the bonding behavior of RC structures should be considered for structural fire engineering design. Today, general information is available about the material deterioration of concrete and rebars at high temperatures. However, research on the effect of bonding between concrete and rebars at elevated temperatures is still limited. Experimental studies on bonding have been conducted in the literature.^3,12−19^ Yağan^20^ stated that the main reason for the carrying capacity decrease of the beam elements is the damage on the concrete and the decrease in the clamping of the concrete reinforcement due to this damage. Khan et al.^21^ reported that bond strength decreases up to 44%, starting from 300 °C.

Özkal et al.^22^ observed serious effects on the tensile properties of bare steel bars after 600 °C, which were exposed to high-temperature effects in the range of 23–800 °C. By examining the effect of high temperatures on adherence, it was stated that the bond strength gradually decreases with the increase in temperature.^5,11−13^

In order to improve fire design provisions, studies investigating the structural behavior of RC members exposed to fire have attracted attention for the last four decades through fire tests and advanced analytical methods.^23,24^ However, fire tests are costly and require too much time. Advanced analytical methods are generally based on the finite element method (FEM).^25^ FEM has been regarded as the most common tool for performing advanced fire-resistant analysis, and necessity of structural performance investigation under fire is significantly required.^26,27^ As a result of the studies on the numerical analysis of RC structures under high temperatures, it has been supported that both the thermal and mechanical behavior of the structures can be reliably feasible.^25,28−40^

To predict the behavior of three-dimensional RC members in fire, Lie^25^ and Terro^41^ developed a finite element analysis approach detailing the thermal and mechanical properties of concrete and steel. It has been observed that numerical analysis including thermal and structural modeling is able to coincide with the experimental results to evaluate the performance of RC structural elements during exposure to fire.^30,33^ Jawdhari et al.^42^ developed a transient thermal-mechanical finite element technique by using ANSYS, which includes two separate but related time-domain simulations of heat transfer on a nonlinear structure. Ozbolt et al.^33^ numerically simulated the behavior of RC. The initial strength of the beams decreased with the increase of the fire exposure time. They also stated in addition to predicting the general response of beams, the model could be an effective tool to numerically investigate a few phenomena that are difficult to observe experimentally. Kodur and Agraval^43^ developed an approach to assess the capacity of RC beams exposed to fire and applied this approach with a detailed numerical model developed in ABAQUS software. It showed that finite element analysis gives more realistic strength results than predicted from the simplified cross-sectional analysis. By using the FEM with ANSYS software, Kada et al.^35^ showed that fire failure of beams would be caused by the web characteristics of the beams in most cases. A detailed three-dimensional temporary thermal finite element analysis was performed by Musmar et al.^36^ to examine the performance of RC beams exposed to fire. The RC beam was exposed to ASTM E119^44^ standard fire on the lower and side surfaces under a time-bound temporary temperature load, while a constant load was applied on the upper surface. Verification of the finite element model is compared with the experimental results performed for similar RC beams under the same conditions. Ryu et al.^37^ exposed the ISO-834^45^ standard fire curve to different RC beams with different loads. After the experimental stage, numerical analysis was performed with the ABAQUS software. Results showed that the higher the load level, the higher the temperature distributions. The temperature distributions can be explained by the crack propagation caused by loading. Failure of the beams developed as the fire temperature increased. Trong et al.^38^ investigated the effects of fire on temperature distribution in the concrete structure using the finite element method by ANSYS software. Based on the law of temperature distribution, it was stated that it is possible to determine the variation of mechanical properties such as elastic modulus, strength, strain, and so forth. Elshorbagy and Abdel-Mooty^39^ investigated the effect of fire parameters on compressive strength of concrete, concrete cover, and lateral stiffness of the beam with detailed numerical finite element models using the ANSYS program and supported their findings with experimental observations.

Previous studies on structural fire behavior provide essential information about parameters affecting the bonding performance at high temperatures and form a solid basis for developing numerical models for interlocking bonds.^46^ Since numerical models are insufficient to interpret the effects of bonding properties between concrete and rebars at high temperatures, most numerical models developed to predict the fire behavior of RC structures are based on the assumption of fully bonded contact.^47^ More research has been required to accurately estimate the impact of fire on structural safety and performance and to identify ways to reduce this impact. Therefore, the primary purpose of this research is to develop robust numerical models for predicting the adherence between concrete and reinforcement under fire conditions and finding a reliable method to simulate the thermal–structural analysis of RC members and evaluate critical parameters affecting the performance of RC structures during exposure to fire loads.

Currently, a limited number of numerical models are available to consider bonding behavior at high temperatures. Several researchers such as Bolmsvik et al.^48^ studied to simulate the adherence between concrete and rebars. However, studies^32,49−51^ simulating the bond-shear relationship under high temperatures are limited. Huang^47^ adopted the CEB-FIP^52^ (1991) adherence model at ambient temperature and considered the deterioration of bond strength at high temperatures using the experimental results generated by Bazant and Kaplan.^53^ Huang’s model^47^ was a significant step forward on evaluating bonding properties in a fire. Hemmaty et al.^54^ considered a nonlinear bond-shear law based on experimental studies between concrete and rebars. They stated that material models could also be used for modeling the bond-shear relationship (with reduced tensile and compressive strength concrete material), and they suggested the actual modeling with COMBIN39 spring elements. As a result, the crack results obtained from four different material models defined for the spring element in ANSYS were compared with the cracks in the samples applied with the tensile test and it was stated that concrete-rebar bonding could be modeled realistically by using appropriate bond-shear laws.

## 3 Finite Element Modeling

Finite element models of the RC beams were developed using ANSYS (Release 18.2)^55^ software. Two types of analysis are needed to model the beam exposed to mechanical and thermal loads. First, thermal analysis is required to calculate temperatures across the beam at any time step. Through this analysis, the fire load was applied in the form of thermal load in addition to the structural loads in the structural analysis. There are two options to conduct two different analyses together. The first option is to apply the result of one analysis as the initial boundary condition for the other analysis, where each analysis type is performed separately. The second option is performing both analyses simultaneously.

The thermal RC element (SOLID70) used in this study does not have the feature of cracking, which prevents observing the cracks in concrete. Additionally, since the steel rebar (LINK180) in a structural analysis has no thermal equivalent, it is not possible to directly perform both analyses simultaneously. For all these reasons, the sequentially combined thermo-mechanical analysis was preferred.^56^ As the temperatures from thermal analysis form the input for structural analysis, thermal analysis is to be carried out first, followed by structural analysis.^57^

The thermal–structural analysis steps in the ANSYS program are shown below

Step 1 Define the beam model for structural analysis.
Step 2 Evaluation of the beam’s final capacity at room temperature
Step 3 Define the beam for thermal analysis without deformation
Step 4 Thermal analysis solution.
Step 5 Structurally changing the analysis and thermal type and defining the structural model.
Step 6 Adding the thermal analysis results.
Step 7 If there is a collapse in the beam strength; evaluation of fire resistance, if there is no collapse formation; evaluation of residual strength by applying mechanical loads.

### 3.1 Elements

#### 3.1.1 Thermal Elements

SOLID70 was used to model the behavior of the concrete in heat transfer problems due to its heat conduction feature. It has eight nodes with one degree of temperature freedom in each node. LINK33 was used to model longitudinal and transverse reinforcements as it can transmit heat between two points. LINK33 is a uniaxial element with one degree of freedom at each node. Both elements can be used in 3D time-independent or 3D time-dependent thermal analysis.

Bond shift modeling was considered in the study to achieve more realistic results in numerical modeling. As the standard approach in structural analysis of RC members, 100% bonding (adherence) is assumed between concrete and rebar. However, this is not possible in actual conditions due to human errors, concrete casting, weather conditions, and so forth. Hence, the COMBIN39 nonlinear spring element was preferred at the coinciding nodes of the concrete and rebars. It is a one-directional spring element that nonlinear force–deformation relations can be defined. COMBIN39 is defined by a nonlinear force–displacement curve and two nodes.^58^ At the same time, different properties can be assigned to the spring element in tension and pressure, and both torsion and longitudinal elongation in 1-, 2-, and 3-dimensional problems could be operated. Uniaxial tensile-pressure characteristic of the element has three degrees of freedom at all joints for the longitudinal extension option. At the same time, these nodes can freely extend in *x*, *y*, and *z* axes.^59^

#### 3.1.2 Structural Elements

SOLID65 was used for modeling concrete because it has plastic deformation, tensile and crushing capabilities in three directions. The element has eight nodes and each node has three translational degrees of freedom (in the *x*, *y*, and *z* axes). The most substantial properties of this element are the processing of nonlinear material properties. LINK180 is a three-dimensional spar element that can be used in uniaxial tension and pressure conditions, which was used to model rebars in concrete. The element has plastic deformation capacity, two nodes, and three translational degrees of freedom at each node (*x*, *y*, and *z* axes). In addition, it is suitable for plasticity, stretching, rotation, and extinction.

### 3.2 Material Characteristics

#### 3.2.1 Thermal Properties

**Figure 1:**
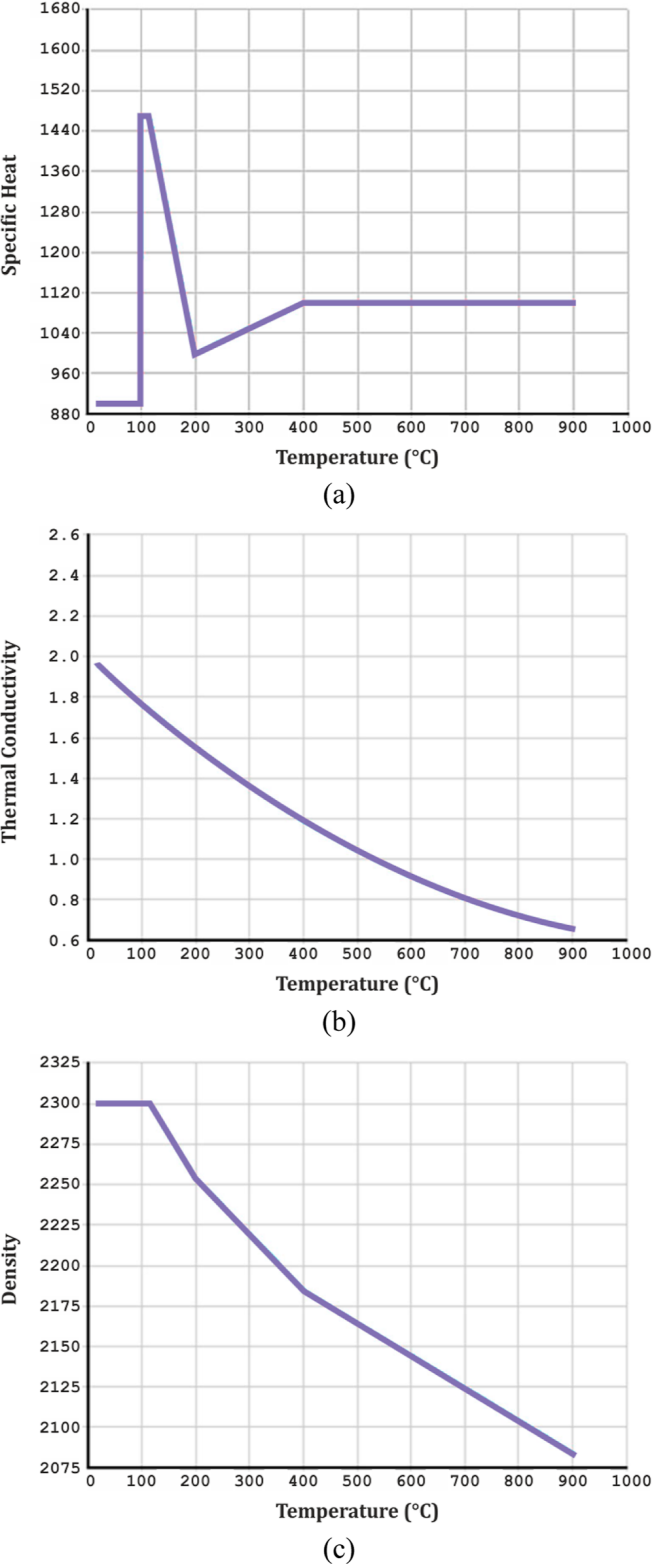
(a) Specific heat; (b) thermal conductivity coefficient; and (c) density curves of the concrete at different temperatures.

The thermal model of the concrete was modeled using SOLID70, and three basic thermal properties (density, specific heat, and thermal conductivity) were defined by calculating from Eurocode 1^60^ and Eurocode 2.^61^ It is expressed in SI and Pa units according to the unit system as classified by ANSYS. The thermal material properties of the concrete are presented in Figure 1.

**Figure 2:**
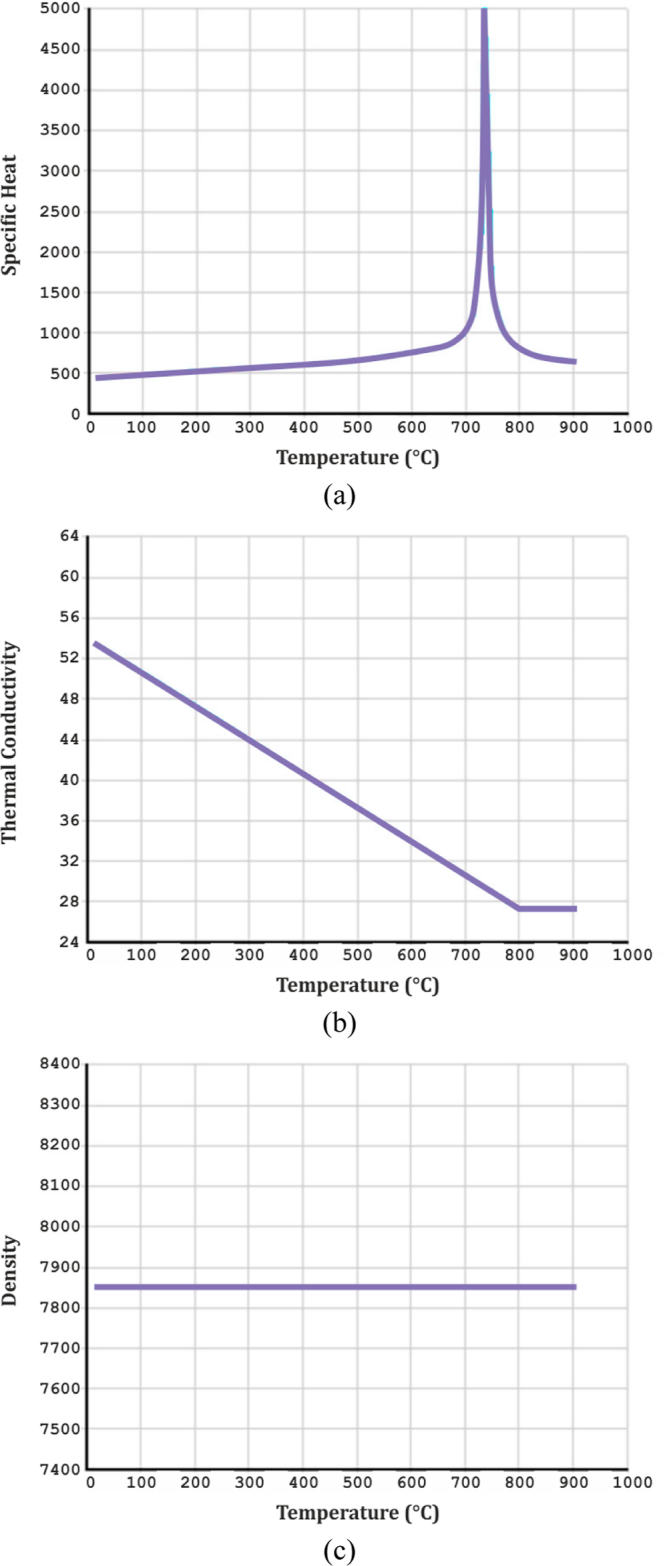
(a) Specific heat; (b) thermal conductivity coefficient; and (c) density curves of steel at different temperatures.

The thermal model of the rebars was modeled using the LINK33 element, and this element was defined by calculating from Eurocode 2^61^ with three basic thermal properties (density, specific heat, and thermal conductivity). Thermal material properties of the steel rebars are shown in Figure 2.

#### 3.2.2 Structural Properties

**Figure 3:**
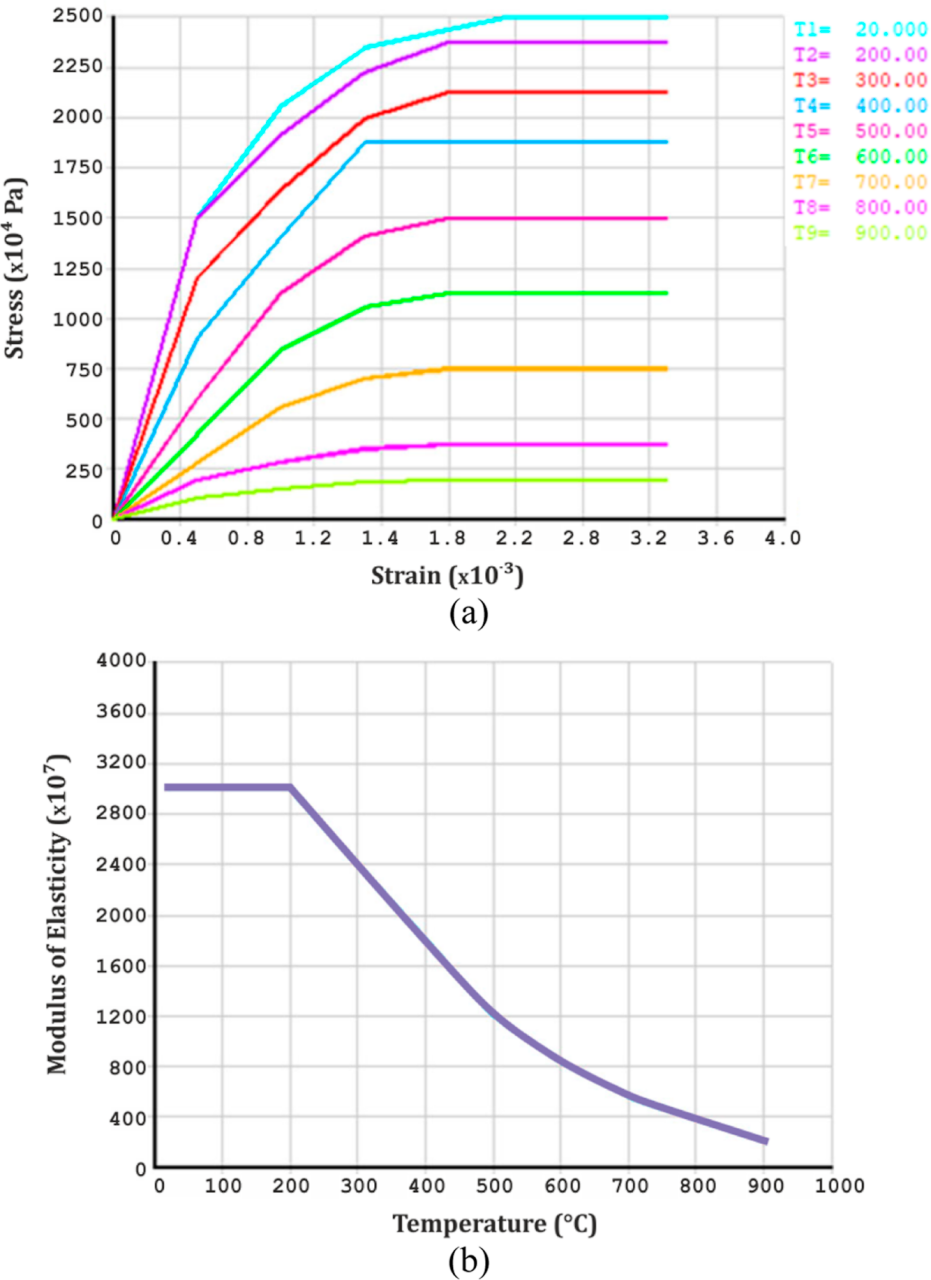
(a) Stress–strain relation and (b) modulus of elasticity variation of concrete at different temperatures.

Stress–strain relationship is generally defined in two parts for concrete and steel rebars. The first part demonstrates the elastic region of the curve by defining the linear isotropic values of the modulus of elasticity in terms of temperature, as explained in Eurocode 2^61^ and Eurocode 3.^62^ The second part demonstrates the plastic region of the curve by defining the multivariate kinematic stiffening values for stress and strain. This process is defined for each temperature range since concrete and steel behave differently at various temperatures. Material properties are shown in Figures 3 and4.

**Figure 4:**
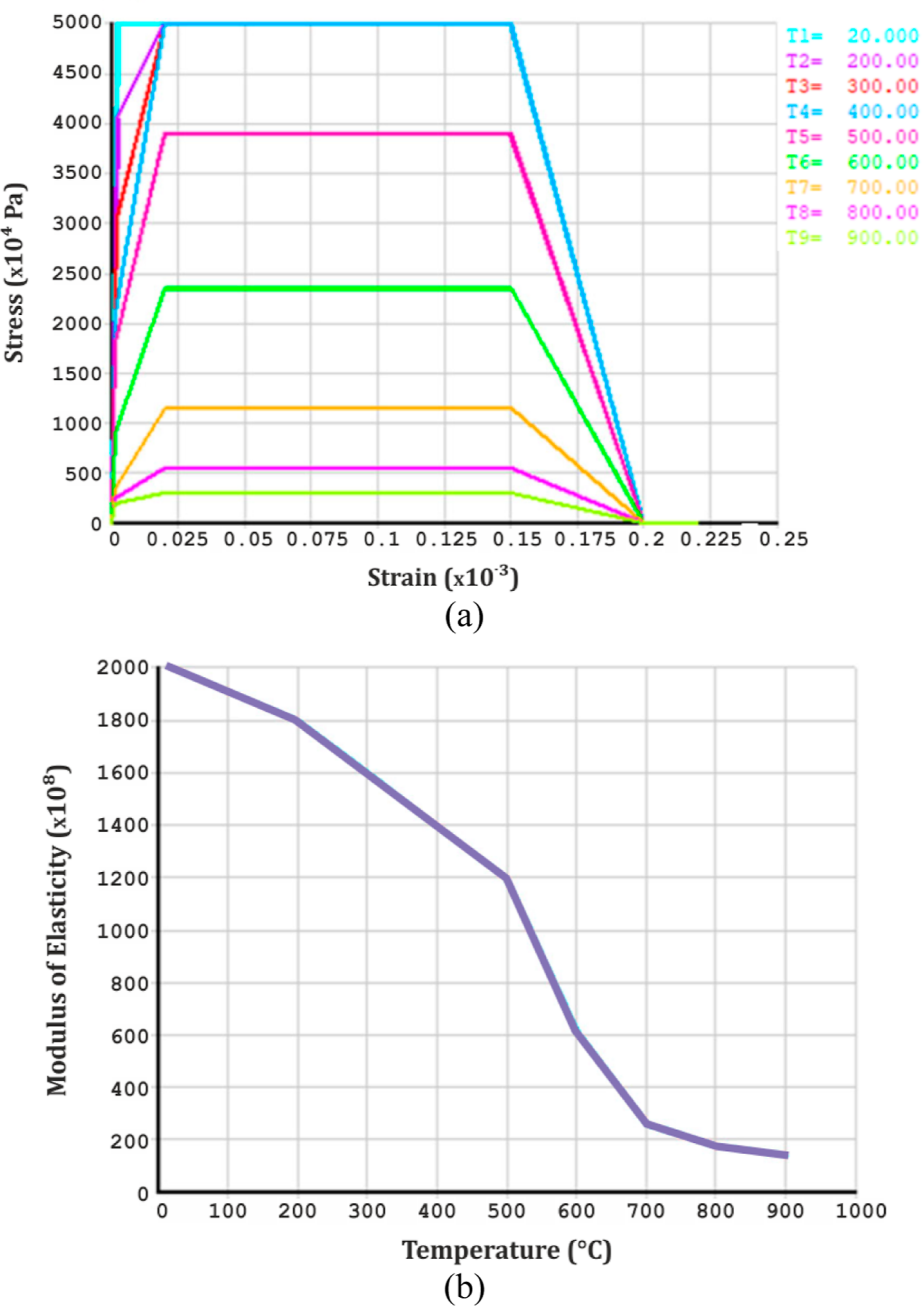
(a) Stress–strain relation and (b) modulus of elasticity variation of steel at different temperatures.

### 3.3 Thermal and Structural Modeling

**Figure 5:**
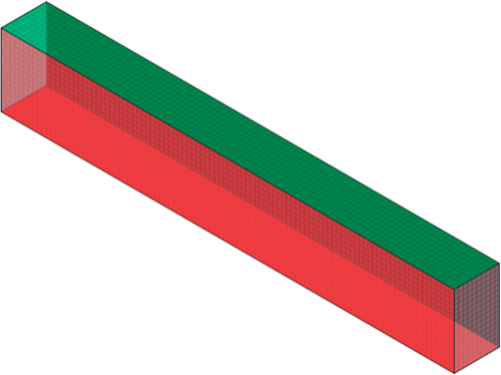
Demonstration of convection load and film coefficients on the beam.

Eurocode regulations^60−62^ provide guidelines for verifying thermal analysis results for RC members exposed to the standard ISO-834^45^ fire curve on one side and all sides. For the improved ANSYS finite element model, film coefficients in surface elements used to apply heat loads in terms of convection were accepted as α_*c*_ = 25 W m^2^ °C^–1^ for surfaces exposed to fire and α_*c*_ = 9 W m^2^ °C^–1^ for surfaces not exposed to fire.^39^ In this study, following the selection of the time-dependent analysis type and initial boundary conditions for the room temperature of 20 °C, fire load is applied to the three surfaces of the beam as shown in Figure 5.

**Figure 6:**
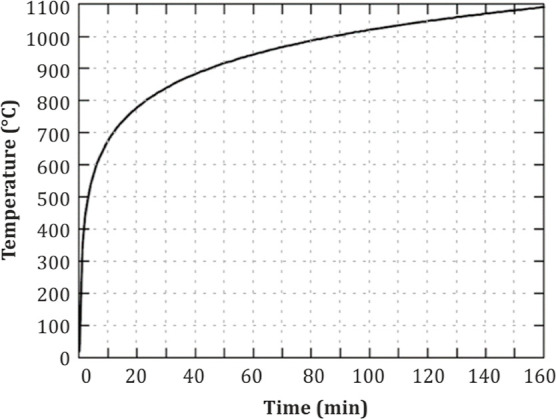
ISO-834 standard fire curve.

To simulate the time-dependent temperature alteration in the beam with the fire phase, a temperature–time curve should be defined as a function. The temperature–time curve that was used in this study is shown in Figure 6. The function of the values used to draw this curve has been obtained from Eurocode 1^60^ and Eurocode 2^61^ according to eq 1.
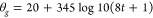
1where θ_*g*_ is the gas temperature in the fire compartment (°C); *t* is the time in minutes; “20” corresponds to the initial temperature (*T*_0_) and if *T*_0_ ≠ 20 °C, it can be changed to another value.

**Figure 7:**
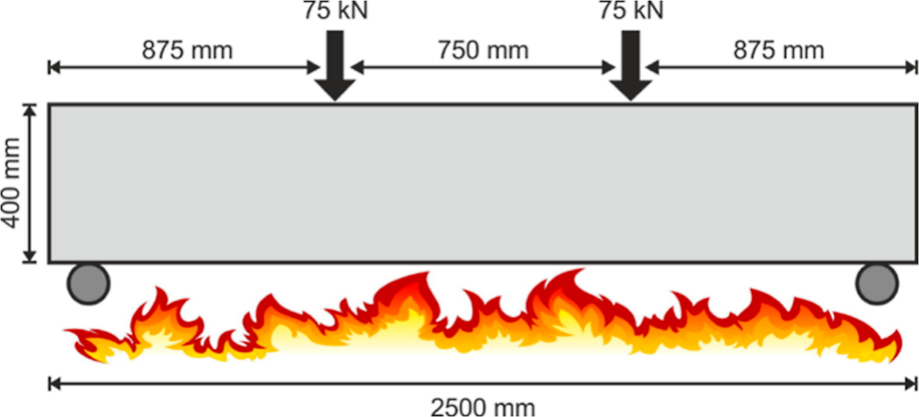
Representation of the thermal-structural loaded RC beam.

After performing thermal analysis, the analysis type is switched to structural, and SOLID70 and LINK33 elements are converted to SOLID65 and LINK180. The fire load applied to three beam surfaces by selecting the time-dependent resolution type is shown in Figure 7 in detail for the RC beam.

While creating the numerical model for thermal and structural analyses, the validation study was initially performed considering experimental research of Kodur et al.^63^ Two different numerical analyses with thermal and structural loads were performed for each of them, and similar results and heat profiles were obtained.

**Figure 8:**
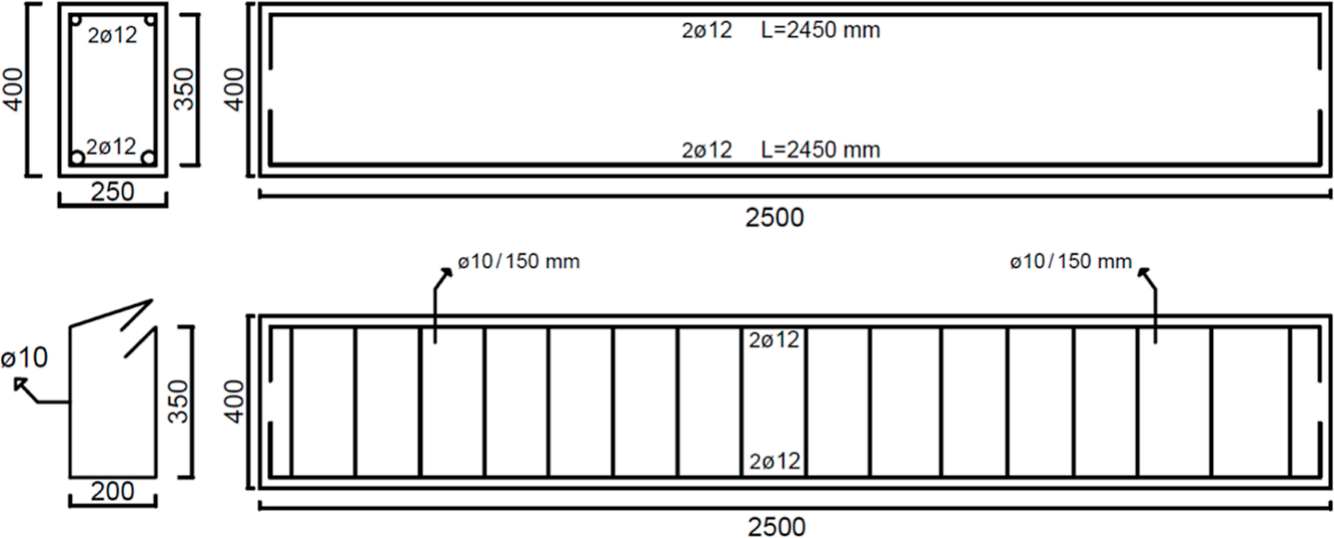
Design details of the RC beam.

Beams were modeled considering the standard strength concrete. The beam dimensions are 2.5 m long, 0.250 m wide, and its total depth equals 0.4 m. The beam has two tensile rebars with 12 mm diameter and two compression reinforcements with 12 mm diameter. Stirrups with 8 mm diameter were placed with 0.15 m spacing. The yield strength of steel is 500 MPa, and the total load given to the beam is vertically 150 kN. The beam geometry, reinforcement spacing, and cross-sectional details are presented in Figure 8 and Table 1.

**Table 1:** Design Chart of the RC Beam

tensile reinforcement	compression reinforcement	shear reinforcement	shear capacity *F*_*u*_^*V*^ (kN)	flexural capacity *F*_*u*_^*M*^ (kN)
2 ϕ 12	2 ϕ 12	16 ϕ 10	50	10

### 3.4 Finite Element Analysis

Within this research, a finite element model of an RC beam was designed to exhibit flexural failure by considering the bonding (adherence) behavior. Finite element modeling of the concrete and rebars has been performed by applying a four-point loading. The beam was subjected to the ISO-834^45^ temperature–time curve, and thermal/structural responses of both beams were evaluated. As soon as the ambient target temperature is achieved for the periods specified in the ISO-834 fire curve, the load-bearing capacities of the beams are determined. Twelve different ambient temperatures were determined, starting with room temperature. The point that makes this study unique is that the ISO-834 fire curve of the ambient temperature allows each temperature to be examined precisely, although the high temperature is reached in a short time. Thermal results were calculated according to the temperature variance over time at each node.

**Figure 9:**
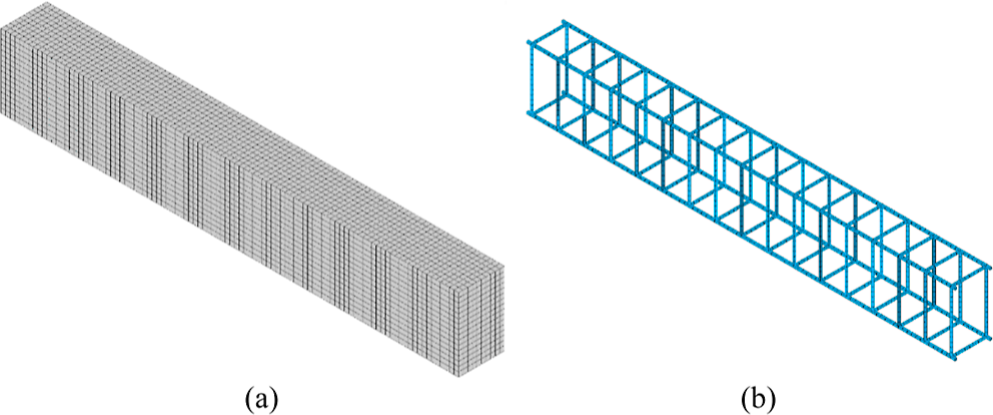
(a) Concrete mesh and (b) rebar mesh for finite element modeling.

Concrete elements have a mesh size of approximately 2.5 cm. Steel elements of longitudinal and transverse rebars were placed, leaving a 2.5 cm concrete cover. Concrete mesh is shown in Figure 9a, and rebar mesh is shown in Figure 9b. After evaluating thermal analysis results, the analysis type was switched to structural analysis. Thermal loads acquired from thermal analysis and structural loads were applied to the RC beams, which were exposed to 12 different ambient temperatures.

**Figure 10:**
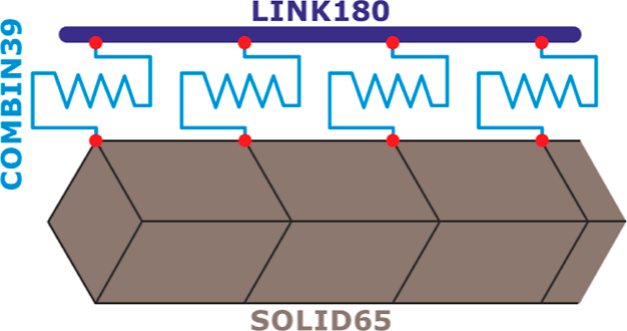
Concrete-steel bond modeling with COMBIN39, SOLID65, and LINK180 elements.

As previously stated for bond modeling, ANSYS assumes 100% adhesion between concrete (SOLID65) and rebars (LINK180). Since this assumption is not practically possible, the COMBIN39 spring element was preferred for bond modeling. Nodes of the concrete and rebar elements were modeled separately, coinciding with each other. An illustration of the applied technique at the coincident nodes is shown in Figure 10. After interlinking nodes with COMBIN39, nodal degrees of freedom are matched in *y* and *z* directions using CP (Couple) command. It was forced to make equal displacements in these directions under lateral loads. In the *x* direction, nodes were intended to displace according to the bond stress-rebar slippage curve given for COMBIN39, and nodes were modeled independently from each other. Concrete-steel bond models of Lowes,^64^ Eligehausen et al.^65^ and Murcia-Delso et al.^66^ are the most basic and widely used approaches.

Bond stress–rebar slippage models at different temperatures are required for thermal analysis. Some of those have been suggested by Hertz,^3^ Diederichs and Schneider,^12^ Morley and Royles,^13^ Haddad et al.,^16^ Özkal et al.,^22^ Huang,^47^ Pothisiri and Panedpojaman,^49^ Raouffard and Nishiyama,^67^ Matsudo and Nishida,^68^ Wang,^69^ Haddad and Shannis,^70^ Khalaf et al.,^71^ and Tariq and Bhargava.^72^

**Figure 11:**
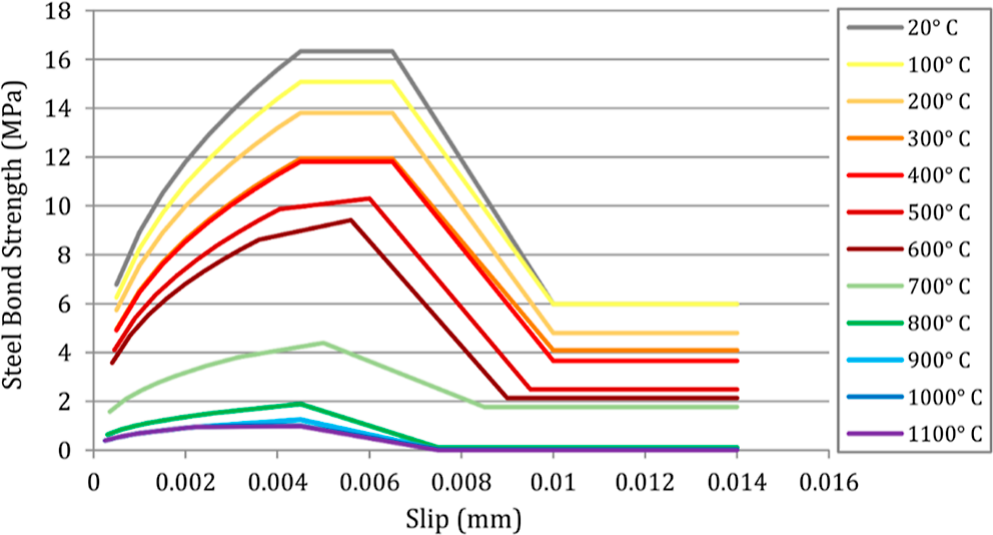
Bond stress–slippage curves in this study for different temperature levels.

In terms of performing a validation study, a bond stress–rebar slippage model was created by averaging and idealizing the suggestions from these previous studies for the different temperature levels as shown in Figure 11. Material properties and specimen dimensions in the previous studies were used as input data to validate the model.

## 4 Discussion of the Results

It is known that in a reinforced concrete structure exposed to fire, the ambient temperature and its own temperature cannot be the same. Since the thermal conductivity coefficient of the concrete is relatively low, concrete transmits the heat more slowly. After the thermal analysis was carried out on RC beams, the maximum temperature values reached through the beam at different ambient temperatures are shown in Table 2.

**Table 2:** Maximum Temperature Levels Through the Analyzed Beams

specimens	target temperature	exposure time to the fire	outer of the beam	interior of the beam
B1	20 °C	0.1 s	20.0 °C	20.0 °C
B2	100 °C	6 s	21.0 °C	20.4 °C
B3	200 °C	18 s	26.0 °C	22.6 °C
B4	300 °C	42 s	37.1 °C	27.6 °C
B5	400 °C	88 s	63.0 °C	39.1 °C
B6	500 °C	178 s	115.4 °C	62.4 °C
B7	600 °C	354 s	206.8 °C	102.0 °C
B8	700 °C	695 s	328.0 °C	157.1 °C
B9	800 °C	1365 s	483.2 °C	227.2 °C
B10	900 °C	2660 s	658.7 °C	312.2 °C
B11	1000 °C	5190 s	836.2 °C	421.2 °C
B12	1100 °C	10800 s	1012.7 °C	586.4 °C

**Figure 12:**
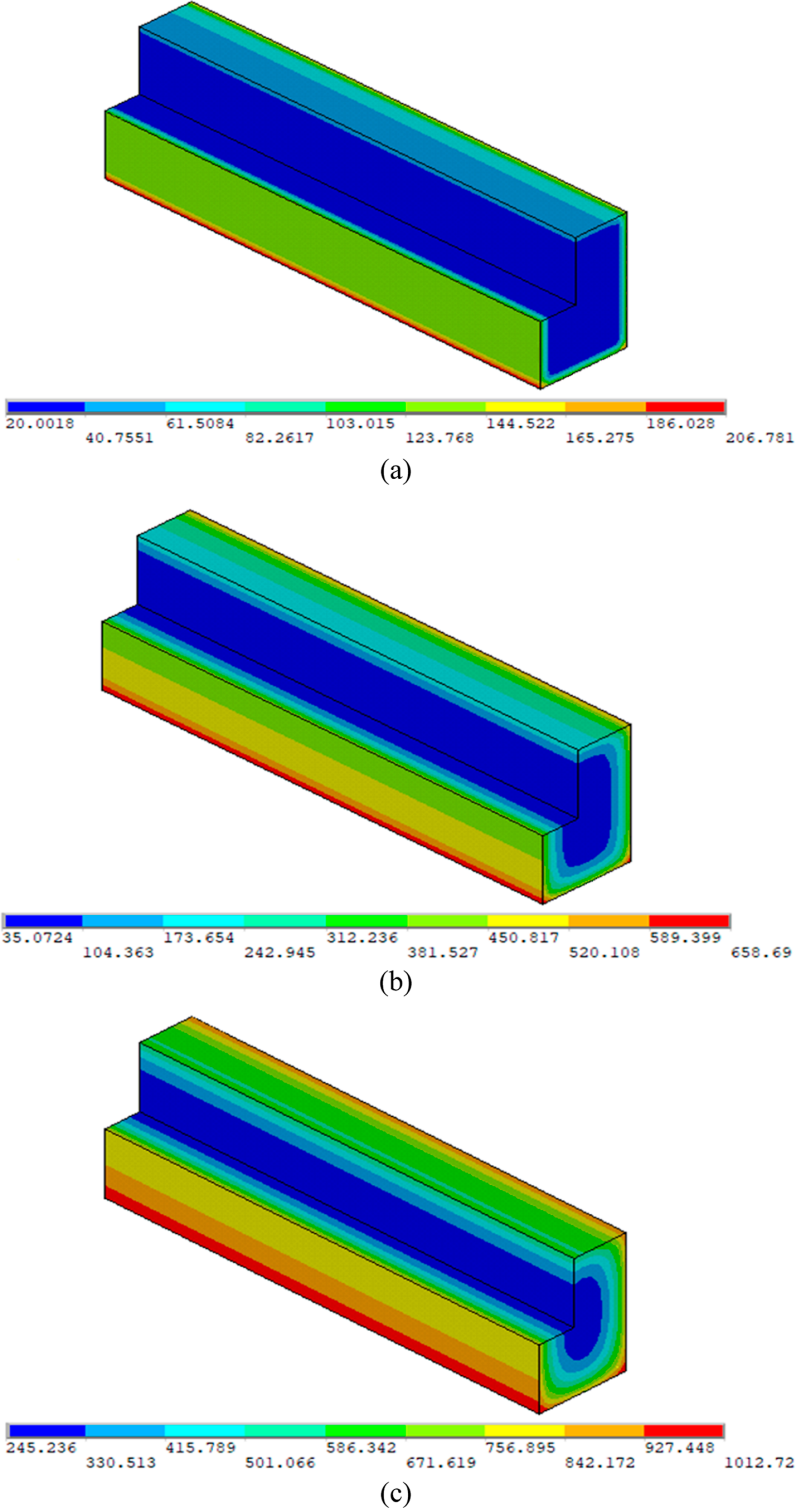
Interior heat profiles of beams subjected to ISO-834^45^ fire at target temperatures of (a) 600; (b) 900; and (c) 1100 °C.

Because the ISO834 fire curve reaches the target temperature in a very short time and thermal conductivity coefficient of the concrete is relatively low, even the outer surfaces of the beam could not reach the ambient temperature. The fact that the heat cannot reach the interior of the concrete in such a short time is clearly seen from the heat distributions of the beams exposed to different temperatures (Figure 12). Although the thermal conductivity coefficient of steel is higher, the rebar temperature did not change much because of the short heating time at which the steel material could not perform heat transmission. From the previous studies, that is, Ellingwood and Lin,^7^ Bamonte and Monte,^73^ and Gao et al.,^50^ supported the mentioned change in the temperature distribution regarding inner and outer sections of the concrete.

**Figure 13:**
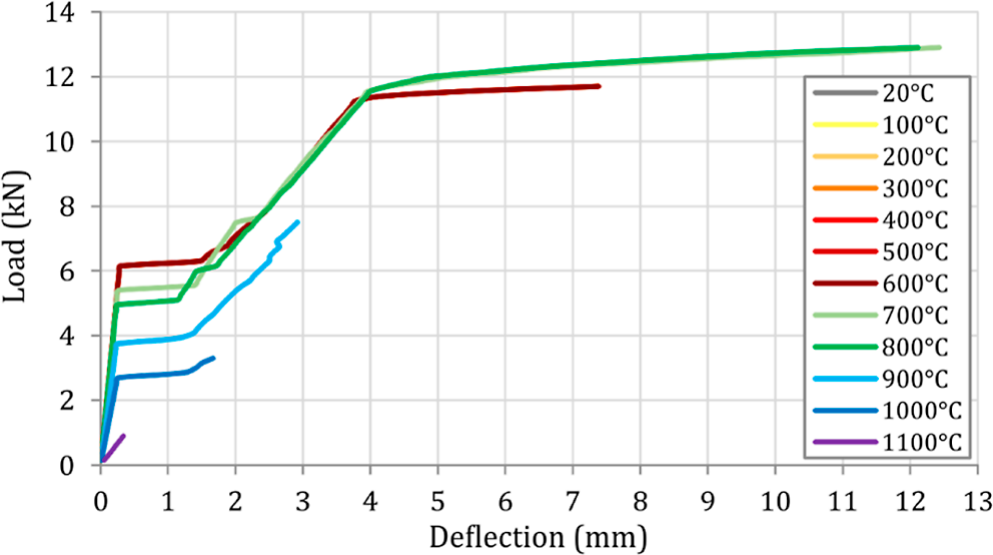
Load–deflection histories of the analyzed beams after exposure to the fire.

**Figure 14:**
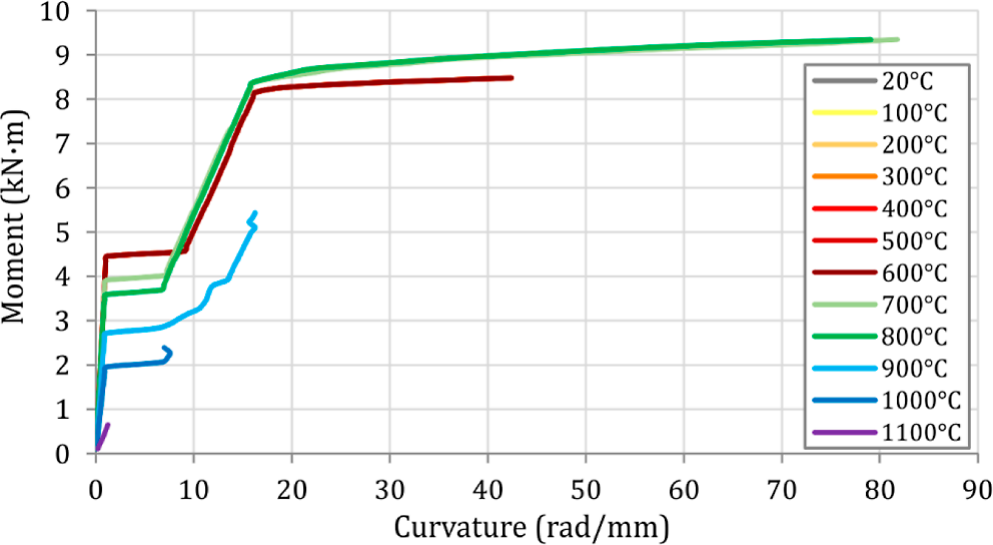
Moment–curvature histories of the analyzed beams after exposure to the fire.

**Figure 15:**
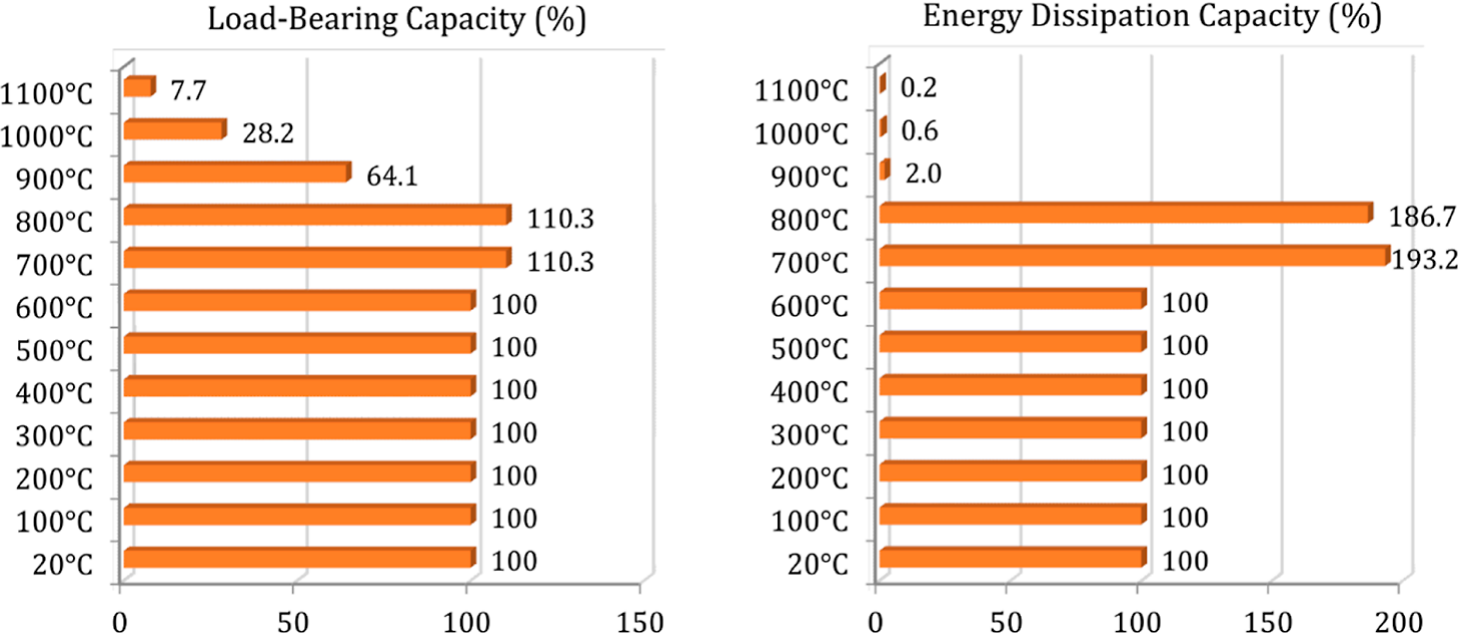
Load-bearing and energy dissipation capacity comparison for the analyzed beams.

**Figure 16:**
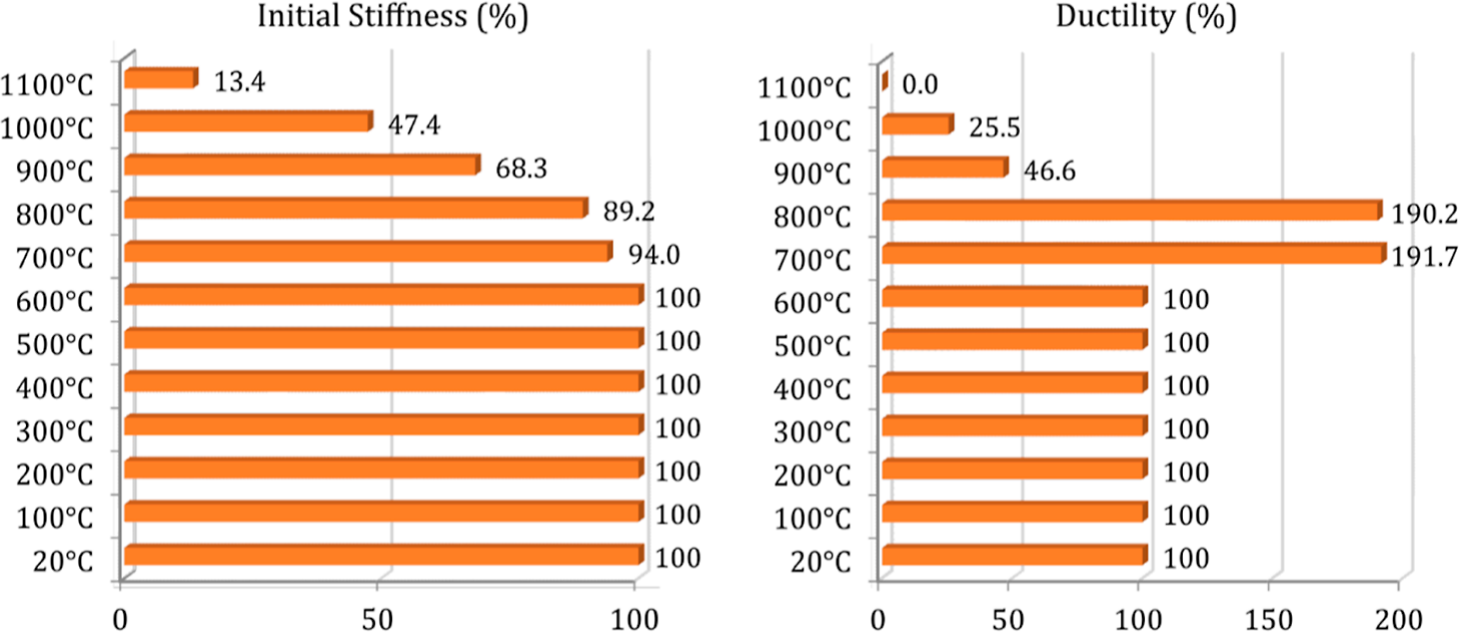
Initial stiffness and ductility comparison for the analyzed beams.

Structural loads were applied on the beams, which were exposed to ISO-834^45^ fire and target temperatures were achieved. Following the thermal and structural analyses, load–deflection and moment–curvature histories of the beams were obtained and are presented in Figures 13 and 14. Figures 15 and 16 also compare load-bearing capacity, energy dissipation capacity, and initial stiffness and ductility values of the analyzed beams. These values were calculated from the load–deflection and moment–curvature histories of the beams at different target temperatures.

When the load–deflection and moment–curvature histories are examined (Figures 13 and 14), it is seen that B1–B7 beams exhibit almost identical behavior and the curves overlap each other. These beams, heated from 20 to 600 °C, did not exhibit any loss on the structural performance and achieved 102 °C maximum temperature at the inner region and 206.8 °C maximum temperature at the outer regions. The main reason for forming similar curves is that the interior of the beams did not reach ambient temperature because of the short exposure time. B8 and B9 beams (700–800 °C) also exhibited similar behaviors with each other but yielded higher structural performance than B1–B7 beams. However, following the level of 900 °C, a significant decrease on the structural performance (residual load-carrying capacity and stiffness) is encountered as the fire duration increases (Figure 15) similar to the findings of Tonidis et al.^74^

The increase for the general structural performance of B8–B9 beams (700–800 °C), in which the concrete achieves the 150–250 °C temperature range, is supported by previous studies. To explain also the structural performance increment after 700 °C and sequential decrease after 900 °C, outcomes of some previous studies could be beneficial. Handoo et al.,^75^ Savva et al.,^76^ and Aydın et al.^77^ indicated that the concrete compressive strength increases with increasing temperature between 100 and 300 °C. On the other hand, Yamazaki et al.^78^ came across an increase in the compressive strength of concrete at around 200 °C, and after that, rapid decreases were observed in strength with the increase in temperature. Xiao and König^79^ stated that when the compressive strength of the concrete is increased from room temperature to 400 °C, they observed a small decrease, and then, a slight increase and a very significant decrease in strength when the temperature reached 400 °C. They stated that only 20% of the initial strength could be preserved at 800 °C. Rashid et al.^80^ stated that there is no significant change regarding the concrete characteristics up to 150 °C, and when the temperature rises above 150 °C, it starts to lose strength. As a result of the study by El-Hawary and Hamoush,^5^ an increase in the bond strength of the samples, which were heated at 100 °C for a short time, was determined. This increase was explained as shrinkage due to the evaporation of water in the concrete and providing a better interlocking, but they stated that it was due to the decrease in adherence with the increase in temperature and heating time at later temperatures. Bingöl and Gül^17^ also stated that the bond strength of the concrete increases until 150 °C.

Concrete is a non-combustible material and is more resistant to fire than other construction materials. However, the compressive, tensile, and bending strength values of the concrete decrease due to elevated temperature. In addition, it is stated in different studies that there may be no deterioration in concrete characteristics up to 200 °C, and even a slight increase in strength. These strength increases are explained by the energy generated by the effect of temperature that causes the unhydrated cement grains to complete their hydration. In addition, it should be considered that sample properties and test conditions are important parameters in determining the behavior of the concrete after high temperatures, and there may be differences between the outcomes.^81^

The critical ambient temperature for the beam was found to be approximately 900 °C considering the B10 beam, which achieved more than 300 °C at the inner region and 600 °C at the outer regions and 36% of the beam strength was lost at this level. Despite the reasonable decrease in load-bearing capacity and initial stiffness, B10 and B11 beams lost 98 and 99.4% of their energy dissipation capacities. Furthermore, the B12 beam (1100 °C) with an internal temperature of nearly 600 °C received excessive damage. The mentioned outcomes are supported by Figures 15 and 16.

When the internal temperature of the beams was between 150 and 250 °C, they had the maximum load-bearing capacities with an increment of nearly 10% as explained in detail. Furthermore, a comparison of energy dissipation capacity and ductility (Figures 15 and 16) represents excessive increments for these specimens alongside the concrete strength increment. This behavior is triggered owing to the increase in concrete compressive strength and correspondingly to the more increment in concrete-rebar bonding performance. As the internal temperature increased above 250 °C (600 °C at the outer regions of the beam and 900 °C ambient temperature), three of these behavioral indicators decreased due to the loss of concrete strength and the initial stiffness. After the increased exposure time to fire and correspondingly to the increase of ambient temperature from 600 °C, the specimens gradually began to lose stiffness. However, a sudden decrease in these indicators was observed due to the excessive deterioration of the concrete cover on the steel reinforcements. As expected, this performance degradation caused the brittle failure of the beams, which is also recognizable from load–deflection and moment–curvature histories (Figures 13 and 14). Ductility coefficient, the basic parameter indicating the level of plastic strain capacity, was also compatible with the concrete strength and decreased gradually after the 900 °C ambient temperature.

## 5 Conclusions

Within the scope of this study, reinforced concrete (RC) beams were designed to exhibit failure by forming flexural cracks, taking into account the different fire durations and temperatures based on the ISO-834^45^ fire curve. Furthermore, an algorithm has been developed by combining nonlinear finite element analysis with thermal analysis. The finite element method makes it easier for researchers to study various structural problems and achieve a solution in a shorter time compared to experimental investigation.

It has been observed that the fire effect increases the load-bearing capacity of the RC beams up to a specific temperature and then decreases that rapidly as the ambient temperature increases. This boundary level is 100 °C for the internal temperature. Therefore, it can be asserted that steel rebars do not have a significant role in heat transmission depending on the volumetric ratio compared to concrete. Hence, the rebar temperature could be considered equal to the concrete temperature at the depth where the reinforcement is located.

The heat profile of the concrete was examined in two ways by taking cross sections from the outer and inner regions. A rapid temperature increase was observed on the outer regions of the beam that was exposed to heating directly. However, heat distribution at the interior regions could not rise above 250 °C until the ambient temperature was 900 °C. The main cause is the sudden increase in temperature in a very short time regarding the ISO-834 fire curve. The environment reaching high temperatures in a very short time caused the concrete to not lose its load-bearing capacity for a while due to the slow heat transmission. Exposure time to elevated temperatures draws more attention to the apparent importance of exposure time to heating in determining the residual strength of RC members, rather than ambient temperature.

The numerical modeling approach in this research is compatible with the results of the previous studies. As shown in the numerical simulation of the reinforced concrete beams, it is clear that the internal surface temperature reached by the beam is more determinant of the bearing capacity than the ambient temperature. At the same time, it was observed that concrete compressive strength and correspondingly the concrete-rebar bonding performance increased when the internal temperature was between 150 and 250 °C and structural deterioration started to occur when it exceeded 300 °C. Therefore, such models can be further used to design and optimize fire protection systems to achieve cost-effective solutions.
